# Cervical Cancer: Community Perception and Preventive Practices in an Urban Neighborhood of Lagos (Nigeria)

**DOI:** 10.1155/2014/950534

**Published:** 2014-02-04

**Authors:** K. O. Wright, O. Aiyedehin, M. R. Akinyinka, O. Ilozumba

**Affiliations:** Department of Community Health and Primary Health Care, Lagos State University Teaching Hospital (LASUTH), Ikeja, Lagos, Nigeria

## Abstract

*Background*. Cervical cancer prevention in developing countries is suboptimal compared with the developed world where there are fewer deaths and improved survival rates. This study describes the perception and preventive practices on cervical cancer by residents of an urban neighborhood of Lagos, Nigeria. *Methods*. A descriptive cross-sectional study was conducted on 317 consecutively recruited consenting participants at a medical outreach using a pretested, interviewer-administered, semistructured questionnaire. Data analysis was done using statistical package for social sciences version 19. Tests of significance were performed using 95% confidence interval with level of significance set at *P* < 0.05. *Results*. The majority of respondents were within 30–49 years of age (46.7%) and female (62.1%) and 70.3% had secondary level education and above. About 37.2% of respondents had heard about cervical cancer with 84.5% of the participants willing to attend a cervical cancer health education program. Among the female respondents, 4.1% had received the HPV vaccine, while 5.1% had undergone a Pap test. Awareness about cervical cancer was significantly higher with increasing age in the total population (*P* < 0.05). *Conclusion*. There is a need to improve awareness of at-risk groups and the menfolk about cervical cancer based on the immense benefit of male involvement in reproductive health matters.

## 1. Introduction

Invasive cervical cancer (ICC) is the leading cause of cancer-related deaths among women in developing countries [1]. It is the second most common cancer among women worldwide with an estimate of over 500,000 new cases and about 275,000 deaths in 2008. About 86% of these cases occur in developing countries representing approximately 15% of female cancers [2, 3].

The incidence of cervical carcinoma in Africa is on the rise. However, the true incidence in many African countries is unknown as there is gross underreporting [1]. Few countries have functioning cancer registries but record keeping is suboptimal. Many of the figures in literature are hospital-based, and these represent a small fraction of women dying from cervical cancer, as most women cannot access hospital care and oftentimes die at home [4]. Mortality rates in developed countries with successful screening programs hardly exceed 5 per 100,000 women. In sub-Saharan Africa in 2002, survival rate was 21% compared with 70% and 66% in USA and Western Europe, respectively [2]. In Nigeria, 80% of the 10,000 women who develop cervical cancer annually die from it [5].

The known primary underlying cause is the humanpapillomavirus (HPV), which is the most common sexually transmitted infection worldwide, and it is estimated that 50% to 80% of sexually active women are infected at least once in their lifetime [6]. Prevention of cancer of the cervix is achievable by preventing HPV infection and ensuring early detection and treatment which significantly reduces its morbidity and mortality. Pap smear test is one of the most reliable and effective cancer screening tests available. About 75% of women in industrialized countries have been screened for cervical cancer in the previous five years, compared to less than 5% in developing countries [7]. In sub-Saharan Africa, few women are ever screened for cervical cancer. Screening coverage in developing countries is low ranging from 2.0% to 20.2% in urban areas and 0.4% to 14.0% in rural areas [8]. Low levels of awareness and poor knowledge of cervical cancer coupled with unavailability and inaccessibility of cervical cancer screening services are responsible for the very small number of women being screened in developing countries [9].

Lagos State has two tertiary and about 22 government owned secondary health care facilities where follow-up and treatment may be accessed for cases of positive Pap smear results. This survey was undertaken to assess community perception and preventive practices on cervical cancer amongst residents of an urban neighborhood of Lagos, Nigeria.

## 2. Materials and Methods

### 2.1. Location of the Study

Lagos State is located in southwestern Nigeria and lies on longitude 3° 24′ E and latitude 6° 27′ N. Lagos State has an estimated population of 15 million people [10]. It is made up of 20 local government areas, and 37 local council development areas (LCDA). Olusosun is located in Onigbongbo LCDA of Lagos and is an area known widely on account of the presence of the largest landfill in Lagos State [11]. The area is presently a commercial and residential area.

### 2.2. Study Design

A descriptive cross-sectional study design was used and the study population was selected among the residents of Olusosun area.

### 2.3. Sample Size Determination

Sample size was established such that an accuracy of 5% could be reached around an observed probability of 25%.

The required sample size was calculated using the formula *Z* ^2^ *pq*/*d* ^2^ [12].

Therefore minimum sample size calculated was 288. A nonresponse rate of 10% (+29) was anticipated; therefore, a total sample size of 317 was obtained.

### 2.4. Sampling Method

A medical outreach (consisting of free medical consultation and basic services) was conducted in June 2013 at Olusosun community by resident doctors undergoing medical specialist (postgraduate) training at a tertiary health institution in Lagos as part of the activities for the annual general week of the resident doctors involving elections, medical mission, and other programs. Residents of the community had been sensitized about the program prior to the Outreach to promote community participation in the event. Consecutive recruitment was done for consenting residents until the sample size was obtained. The inclusion criterion is the condition that the respondents must be 18 years of age or above.

### 2.5. Survey Instruments

A structured interviewer-administered questionnaire developed by the researchers (based on literature search) was used for data collection. This instrument was pretested in a different community close to the tertiary health institution to exclude ambiguities and administered by research assistants who had a day training to ensure lexical coherence as well as efficient and effective discharge of duties.

The questionnaire sought information on sociodemographic details, perception, and preventive practices on cervical cancer.

### 2.6. Data Analysis

Analysis was done using Statistical Package for social sciences (SPSS) version 19. Tests of significance were performed using a 95% confidence interval, and the level of significance was set at *P* < 0.05. Bivariate analysis was done to determine associations between variables. Outcome measures include perception of cervical cancer, knowledge of preventive practices, and proportion of respondents who had engaged in preventive practices.

Ethical clearance was obtained from the research and ethics committee and informed consent was obtained from each participant at the point of data collection.

### 2.7. Study Limitation

Lagos State is divided into 20 local government areas (LGAs) with a breakdown of 16 urban and 4 rural LGAs. The study was conducted in one of the LGAs. The findings might not be representative of the entire state (rural and urban combined), but it is fairly representative of the urban communities of Lagos.

## 3. Results

See Tables 1, 2, 3, 4, 5, 6, 7, and 8.

## 4. Discussion

The results from this study show that just over a third of respondents (37.2%) had heard about cervical cancer, with television (39%) being the commonest source of the information. This is quite low when compared to a cross-sectional survey of 650 women conducted in London, which showed that 76.2% of the participants perceived cervical cancer to be a common disease and were also aware of the risk factors for the disease [13]. Similarly, a Yemeni study revealed that most respondents (80.6%) had heard about cervical cancer with a quarter of the respondents (26.4%) accessing this information from television [14]. In Africa, a study in Kenya, to assess knowledge and practices about cervical cancer and Pap smear testing among cervical cancer and noncancer patients, found that 51% of the respondents were aware of cervical cancer which is also higher than that obtained in the present study [15].

Sub-Saharan Africa which appears to be one of the most affected regions also has the lowest awareness rates about cervical cancer, as even lower rates were reported in Cameroon where a study to assess the knowledge of cervical cancer showed that only 28% had prior knowledge of cervical cancer and another study in Ibadan among women aged 20 to 65 years attending the general outpatient unit of a department in a tertiary hospital found that only 15% of the respondents had heard of cervical cancer [4, 16]. Among rural women in a study conducted in Osun State of Nigeria, about 39.2% of the women had heard about cervical cancer which is also very similar to the findings in the present study [17].

Suboptimal awareness of cervical cancer is further demonstrated in the present study by the fact that only 5.1% of respondents who had heard of cervical cancer understood that HPV infection has been strongly implicated in its causality. Correspondingly, none of the respondents in a study conducted in Malaysia had heard of the HPV [18]. The low awareness in these countries that have a greater share of cervical cancer deaths than high income countries is worrisome.

In the midst of this, however, a favorable finding is that 84.5% of respondents are willing to attend a cervical cancer heath education program. This gives an indication that the population is willing to learn about cervical cancer and its prevention in at-risk groups.

Based on the minimal proportion of cervical cancer, informed respondents, it is not surprising that over 90% of the study participants were uninformed on ways of preventing cervical cancer with only 6% of all respondents having heard of the HPV vaccine. These are similar to the finding in a cross-sectional survey among college women in a university in Ghana which showed that only 7.9% were aware of the link between humanpapillomavirus and cervical cancer [19]. Findings from the present study are also similar to those reported from an intervention study conducted among market women in Lagos to evaluate the effect of health education on awareness and uptake of cervical cancer screening where only 6.9% and 12.0% of respondents in the study and control groups had heard about Pap smears at baseline [20]. These figures are quite low when compared with findings from the study conducted in Yemen where a great majority (70%) of respondents were correctly informed that cervical cancer is preventable. Proportions of 59% and 18% of participants in that study knew that routine screening by Pap smear and vaccination against HPV infection are some methods for the prevention and control of cervical cancer [14].

Knowledge generally does not necessarily translate to practice in numerous cases as has been observed on certain occasions. For instance, despite the better awareness observed about cervical cancer prevention in the Yemen study, preventive practices were low with only 19 (7%) of all those knowledgeable about cervical cancer reported having received cervical cytology testing (Pap smear test) [14]. This is comparable to findings in the present study where among the respondents, who had heard of cervical cancer, only 8.5% had undergone a Pap smear test and 6.8% had received the HPV vaccine. Some limiting factors may include cost, availability of vaccines, and screening services as well as accessibility. Contrarily, findings in developed countries such as UK showed that 80.5% of women in a study in London had undergone at least one Pap smear test with 71.5% reporting regular smear tests (every 3–5 years) [13].

Furthermore, screening practices were reported to be poor in a South African survey where in spite of good knowledge on cervical screening and readily accessible facilities with available screening services, the majority of women (87%) from higher social and educational backgrounds did not undergo cervical screening [21]. In Nnewi, Nigeria it is interesting to observe that, among female health care workers who are expected to uptake innovative promotive and preventive health practices, only 5% had ever had a Pap smear done [22].

## 5. Conclusion

The findings in the present study indicate that although awareness of cervical cancer and its prevention appear poor, there was a willingness to learn about this preventable cancer by all respondents. This can form the basis for improving awareness of at-risk groups and other stakeholders including the menfolk particularly in the light of the immense benefit of male involvement in reproductive health matters in Lagos, Nigeria, and Africa as a whole. It is therefore very important to develop ways of ensuring that the message about cervical cancer and its prevention spreads to the grassroots across Nigeria and sub-Saharan Africa to reduce the avoidable morbidity and mortality. In addition, primary and secondary prevention need to be emphasized in terms of health education, HPV vaccination, and cervical screening services which should also be made readily available and accessible in both rural and urban areas.

## Figures and Tables

**Table 1:** Demographic details.

Variable	Frequency (%) (*n* = 317)
Age (years)	Mean: 41.1 Std. dev: 15.4
<30	79 (24.9)
30–49	148 (46.7)
50–69	68 (21.5)
70–89	19 (6.0)
No response	3 (0.9)
Gender	
Male	120 (37.9)
Female	197 (62.1)
Marital status	
Single	80 (25.2)
Married	199 (62.8)
Divorced	3 (0.9)
Widowed	29 (9.1)
Separated	4 (1.3)
No response	2 (0.6)
Educational status	
No formal	28 (8.8)
Primary	66 (20.8)
Secondary	163 (51.4)
Tertiary	58 (18.3)
No response	2 (0.6)
Employment status	
Student	28 (8.8)
Employed	56 (17.7)
Self-employed	194 (61.2)
Home maker	13 (4.1)
Retired	16 (5.0)
Applicant	10 (3.2)

Almost half (46.7%) of respondents were aged 30–49 years. The majority of respondents were female (62.1%) and married (62.8%), and over half of them had secondary school education (51.4%) and were self-employed (61.2%). The mean age was 41.1 ± 15.4.

**Table 2:** Knowledge of cervical cancer.

Variable	Frequency (%)
Heard of cervical cancer	(*n* = 317)
Yes	118 (37.2)
No	199 (62.8)
Risk factor for cervical cancer	(*n* = 118)
HIV	10 (8.5)
HPV	6 (5.1)
Bacteria	17 (14.4)
Do not know	85 (72.0)
1st source of information about cervical cancer	(*n* = 118)
TV	46 (39.0)
Radio	36 (30.5)
Magazine	6 (5.1)
News	7 (5.9)
Health talk	22 (18.6)
Others	1 (0.8)
Knowledge of anyone with cervical cancer	(*n* = 118)
Yes	22 (18.6)
No	96 (81.4)
Have been recommended for screening	(*n* = 118)
Yes	25 (21.2)
No	93 (78.8)
Early detection of cervical cancer is helpful	(*n* = 317)
Yes	196 (61.8)
No	13 (4.1)
Do not know	108 (34.1)

About a third of respondents (37.2%) had heard about cervical cancer prior to the survey. Among these, only 5.1% of the respondents identified human papilloma virus (HPV) as the major risk factor for cervical cancer. The commonest source of information about cervical cancer was via television (39.0%), while 18.1% of respondents knew someone who had had the disease. Despite the fact that only 37.2% had heard of cervical cancer, over half of the respondents (61.8%) were aware of the importance of early detection.

**Table 3:** Attitude to a cancer educational program.

Variable	Frequency (%)
Willingness to attend a cancer health education program	(*n* = 317)
Yes	268 (84.5)
No	29 (9.1)
Do not know	20 (6.3)
Reasons for willingness	(*n* = 268)
More knowledge	227 (84.7)
Better support to partner	29 (10.8)
Others	12 (4.5)
Reason(s) for unwillingness or uncertainty	(*n* = 49)
Embarrassing	4 (8.2)
Women affair	21 (42.9)
Do not know	24 (48.9)
Willingness to allow a male health worker to screen wife (male respondents only)	(*n* = 120)
Yes	94 (78.3)
No	16 (13.3)
Do not know	10 (8.3)

The majority of respondents (84.5%) were willing to attend a cervical cancer health education program for the main reason of acquiring more knowledge (84.7%), while 42.9% of those unwilling to attend felt it was a women's affair. Over three-quarters (78.3%) of the male respondents expressed willingness to allow a male health worker screen their wives.

**Table 4:** Cervical cancer preventive practices.

Variable	Frequency (%)
Heard of ways to prevent cervical cancer	(*n* = 317)
Yes	26 (8.2)
No	172 (54.3)
Do not know	119 (37.6)
Preventive measures known (multiple responses)	(*n* = 26)
Pap smear	17 (65.4)
Vaccination	7 (26.9)
HPV testing	2 (7.7)
Heard of HPV vaccine	(*n* = 317)
Yes	19 (6.0)
No	196 (61.8)
Do not know	102 (32.1)
Recipients for the vaccine	(*n* = 317)
10–25 yrs	8 (2.5)
>25 yrs	7 (2.2)
Do not know	302 (95.3)
Consider vaccination of daughter with HPV vaccine	(*n* = 317)
Yes	199 (62.8)
No	17 (5.4)
Do not know	101 (31.8)
Amongst those aware of cervical cancer, receiving HPV	(*n* = 118)
Yes	8 (6.8)
No	110 (93.2)
If female, received HPV vaccination	(*n* = 197)
Yes	8 (4.1)
No	129 (65.5)
Do not know	60 (30.4)
Amongst those aware of cervical cancer, doing Pap smear	(*n* = 118)
Yes	10 (8.5)
No	108 (91.5)
Had a Pap smear done before	(*n* = 197)
Yes	10 (5.1)
No	127 (64.5)
Do not know	60 (30.4)
Reasons for not having a Pap smear done (multiple responses)	(*n* = 127)
Not at risk	13 (10.2)
Partner will not allow	2 (1.6)
Never heard of it	94 (74.0)
No time	4 (3.1)
Afraid	1 (0.8)
Not sexually active	1 (0.8)

About 8.2% of all respondents had heard of preventive methods for cervical cancer, and among these, about a quarter (26.9%) were aware of vaccinations. Six percent of all respondents had heard of the HPV vaccine, while 2.5% identified the 10–25 year age group as the appropriate recipients for the vaccine. Over half of all respondents (62.8%) would consider vaccination of their daughters with the HPV vaccine. Among the female respondents, 4.1% had received the HPV vaccine, while 5.1% had had a Pap smear before. In about three-quarters (74.0%) of those who had never had Pap smears done this is because they had never heard of it before.

**Table 5:** Awareness of cervical cancer by gender and age.

	Heard of cervical cancer			Test of sig.	*P* value
	Yes	No	Total		
*Gender *					
Male					
<30	7 (29.2)	17 (70.8)	24 (100)	*χ* ^2^ = 5.5	**0.138**
30–49	20 (37.0)	34 (63.0)	54 (100)		
50–69	20 (57.1)	15 (42.9)	35 (100)		
70–89	2 (33.3)	4 (66.7)	6 (100)		

Total	49 (41.2)	70 (58.8)	119 (100)		

Female					
<30	11 (20.0)	44 (80.0)	55 (100)	*χ* ^2^ = 13.33	**0.004**
30–49	36 (38.3)	58 (61.7)	94 (100)		
50–69	18 (54.5)	15 (45.5)	33 (100)		
70–89	2 (15.4)	11 (84.6)	13 (100)		

Total	67 (34.4)	128 (65.6)	195 (100)		

Total					
<30	18 (22.8)	61 (77.2)	79 (100)	*χ* ^2^ = 18.99	**0.001**
30–49	56 (37.8)	92 (62.3)	148 (100)		
50–69	38 (55.9)	30 (44.1)	68 (100)		
70–89	4 (21.1)	15 (78.9)	19 (100)		

Total	116 (36.9)	198 (63.1)	314 (100)*		

*Respondents who did not disclose their ages (3).

Awareness about cervical cancer was significantly higher with increasing age (*P* < 0.05) in female and total population.

**Table 6:** Willingness to attend a cervical cancer educational program by gender.

	Attend program			Total
	Yes	No	Do not know	
Gender				
Male	91 (75.8)	21 (17.5)	8 (6.7)	120 (100)
Female	177 (89.8)	8 (4.1)	12 (6.1)	197 (100)

Total	268 (84.5)	29 (9.1)	20 (6.3)	317 (100)

Pearson Chi-Square = 18.105^a^, Dof = 3, and *P* value = **0.000**.

In comparison with the male respondents, female respondents were significantly more willing to attend a cervical cancer educational program. (*P* < 0.05).

**Table 7:** Willingness to attend a cervical cancer educational program by educational status.

	Attend educational program			Total	Test of significance	*P* value
	Yes	No	Do not know			
*Gender *						
Male						
Educational status						
No formal	3 (60.0)	1 (20.0)	1 (20.0)	5 (100)	Fishers exact = 6.056	0.868
Primary	21 (80.8)	4 (15.4)	1 (3.8)	26 (100)		
Secondary	46 (76.7)	10 (16.7)	4 (6.7)	60 (100)		
Tertiary	21 (75.0)	6 (21.4)	1 (3.6)	28 (100)		

Total	91 (76.5)	21 (17.6)	7 (5.9)	119 (100)		

Female						
Educational status						
No formal	19 (82.6)	3 (13.0)	1 (4.3)	23 (100)	Fishers exact = 8.969	0.312
Primary	36 (90.0)	0 (0.0)	4 (10.0)	40 (100)		
Secondary	93 (91.2)	3 (2.9)	6 (5.9)	102 (100)		
Tertiary	27 (90.0)	2 (6.7)	1 (3.3)	30 (100)		
No resp.	2 (100)	0 (0.0)	0 (0.0)	2 (100)		

Total	177 (89.8)	8 (4.1)	12 (6.1)	197 (100)		

Total						
No formal	22 (78.6)	4 (14.3)	2 (7.1)	28 (100)	Fishers exact = 10.964	0.810
Primary	57 (86.4)	4 (6.1)	5 (7.6)	66 (100)		
Secondary	1139 (85.8)	13 (8.0)	10 (6.2)	162 (100)		
Tertiary	48 (82.8)	8 (13.8)	2 (3.4)	58 (100)		
No resp.	2 (100)	0 (0.0)	0 (0.0)	2 (100)		

Total	1268 (84.8)	29 (9.2)	19 (6.0)	316 (100)		

Educational status was not significantly associated with the willingness to attend a cervical cancer educational program. (*P* > 0.05).

**Table 8:** Permission for wife of male respondents to be screened by male health worker(s).

	Permission to be screened			Total	Test of significance	*P* value
	Yes	No	Do not know			
*Gender *						
Male						
Age in four categories						
17–29	17 (70.8)	7 (29.2)	0 (0.0)	24 (100)	Fishers exact = 17.460	0.063
30–49	41 (80.4)	8 (15.7)	2 (3.9)	51 (100)		
50–69	30 (90.9)	0 (0.0)	3 (9.1)	33 (100)		
70–89	5 (83.3)	1 (16.7)	0 (0.0)	6 (100)		

Total	93 (81.6)	16 (14.0)	5 (4.4)	114 (100)		

There was no statistically significant difference among the various age groups of male respondents with respect to permitting their wives to be screened by a male health worker.

## Appendix

### A. Cervical Cancer: Community Perception and Preventive Practices in an Urban Neighborhood of Lagos (Nigeria)

Dear Respondent,

Cervical cancer is a common female condition throughout the world. This questionnaire is aimed at assessing your understanding and preventive practices regarding cervical cancer. Please be assured that all your responses will be kept strictly confidential.

This is an anonymous questionnaire which does not require your name or identity.

Participation in this study is voluntary, and a refusal to participate will not attract any sanctions.

Please answer the entire questions to the best of your ability and as truthfully as possible.

Your responses will assist the researchers in the prevention and control of this condition.

A research assistant is available to help you in filling the questionnaire.

□ By checking this box I agree that I have read the above and agree to participate in this study.

Thank you for participating.

#### A.1. Sociodemographic Details

(1) Age: ____________ (in years)
(2) Marital status:
  Single □
  Married □
  Divorced □
  Widowed □
  Separated □
  Others ____________ (please specify)
(3) Educational status:
  No formal □
  Primary □
  Secondary □
  Tertiary □
(4) Gender:
  Male □
  Female □
(5) Employment status:
  Student □
  Employed □
  Self-employed □
  Home maker □
  Retired □
  Applicant □

#### A.2. Perception and Preventive Practices on Cervical Cancer

(6) Have you ever heard of cervical cancer?
  Yes □
  No □
  I do not know □
  If yes,
(7) Cervical cancer is caused by
  Human immunodeficiency virus (HIV) □
  Humanpapillomavirus   (HPV) □
  Bacteria □
  Do not know □
(8) Where did you learn about cervical cancer (tick all that apply)
  TV □
  Radio □
  Magazine □
  News □
  Health talk □
  Others ____________ (pls specify)
(9) Do you know anyone with a history of cervical cancer?
  Yes □
  No □
  Dnt know □
(10) Do you think it is helpful to detect Cervical Cancer early?
  Yes □
  No □
  Do not know □
(11) Has anyone ever recommended that you should get tested or screened for cervical cancer?
  Yes □
  No □
  I do not know □
(12) Will you be willing to attend a cancer health education program on women's health?
  Yes □
  No □
  I do not know □
(13) If yes, why?
  More knowledge □
  Better support to partner □
  Others ____________ (Please specify)
(14) If No, why?
  Embarrassing □
  Women affair □
  Do not know □
(15) If you are male, would you allow your partner to be screened/tested by a male health care provider?
  Yes □
  No □
  I do not know □
(16) Have you heard of ways to prevent cervical cancer?
  Yes □
  No □
  I do not know □
(17) Which preventive measures do you know for cervical cancer? (check all that apply)
  Pap smear □
  Vaccination □
  HPV testing □
  Drugs □
  Others ____________ (please specify)
(18) Have you heard of the humanpapillomavirus (HPV) vaccine?
  Yes □
  No □
  I do not know □
(19) If yes, who are those who should receive this vaccine?
  <10 years □
  10–25 years □
  >25 years □
(20) If you have a daughter, would you consider vaccinating her with the HPV vaccine?
  Yes □
  No □
  I do not know □
(21) If you are female, have you been vaccinated with the HPV vaccine?
  Yes □
  No □
  I do not know □
(22) Have you had a Pap smear done before?
  Yes □
  No □
  I do not know □
(23) If no, why?
  Not at risk □
  Cost □
  Partner will not allow □
  Never heard of it □
  No time □
  Afraid □
  Not sexually active □
  Poor health worker attitude □
